# Changes to Czech Corporate Restructuring Laws During the COVID-19 Pandemic: A Comment

**DOI:** 10.1007/s40804-023-00275-5

**Published:** 2023-03-15

**Authors:** Petr Sprinz

**Affiliations:** Counsel, Allen & Overy, Prague, Czech Republic

**Keywords:** Extraordinary moratorium, Suspension of loan repayments, Corporate insolvency filing, Insolvency reliefs, Financial distress, Creditors’ insolvency petition ban

## Abstract

The author briefly comments on various measures undertaken in order to mitigate the effects of the extraordinary situation in connection with the pandemic of SARS-CoV-2 and seeks to put them into the context of the available data. In this connection, the paper mainly focuses on corporate insolvency filings, extraordinary moratoria and suspension of loan repayments. The author also briefly describes the future outlook.

## Introduction

The global pandemic of SARS-CoV-2 brought about unusual challenges to health, business and other areas. The Czech Republic was no exception to this. Jan Lasak describes various measures that were undertaken in order to mitigate the effects of the extraordinary situation in spring 2020.^1^ I will add my view mainly from my perspective as a restructuring and insolvency practitioner focused on corporate debt issues and as a member of the legislative committee at the Ministry of Justice of the Czech Republic during the relevant period. In this context, I will also focus on relevant data and my reading thereof.

Although Czech laws are formally stringent when it comes to potential negative consequences of a failure to file an insolvency petition in time, it appears that businesses enter insolvency proceedings only as a last resort, sometimes having already been at the stage of factual insolvency for several years.^2^ With the mandatory or forced closure of many business premises during the pandemic, entrepreneurs faced huge cash-flow constraints. Suddenly many of them considered insolvency options and used to call their counsel a few times a week to discuss whether they were under a duty to file an insolvency petition.

## Selected Measures with the Aim to Help Businesses

**Fig. 1:**
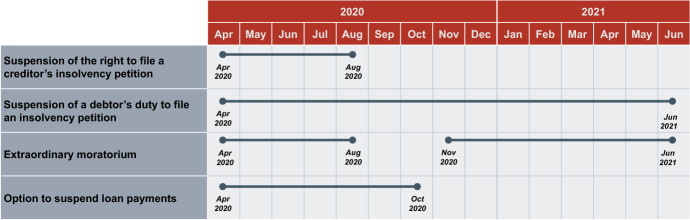
Overview of selected measures and timeframes

In order to alleviate the position of entrepreneurs, the Czech legislature brought about, *inter alia*, several measures, including suspension of the duty to file an insolvency petition, suspension of creditors’ right to file an insolvency petition, introduction of an extraordinary moratorium^3^ and an option to suspend loan payments.^4^ An overview of the selected measures together with their timeframes is given in Fig. 1.^5^

## Corporate Insolvency Filings

**Fig. 2:**
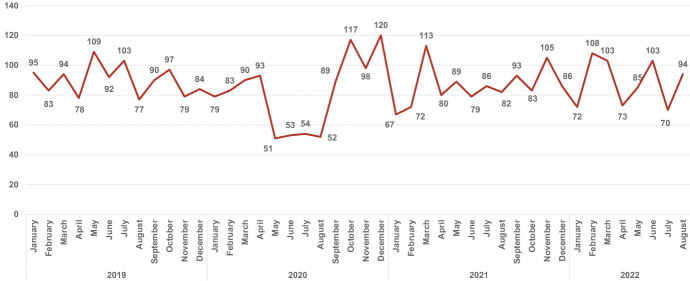
Statistics on corporate insolvency filings. Source: Surveilligence, s.r.o. (data provided to the author on a private basis)

As can be seen from the statistics on corporate insolvency filings in Fig. 2, there was indeed a significant drop in new insolvency filings until the end of summer 2020. Since it was impossible for creditors to initiate insolvency proceedings, all those filings are based on debtors’ insolvency petitions. After the lapse of the creditors’ insolvency petition ban, corporate insolvency filings slightly increased for a few months. However, no huge wave of insolvency filings could be observed. The current energy crisis, together with other setbacks described in Sect. 6 below, has nevertheless put huge pressure on the business sector in the Czech Republic.

## Extraordinary Moratoria

**Fig. 3:**
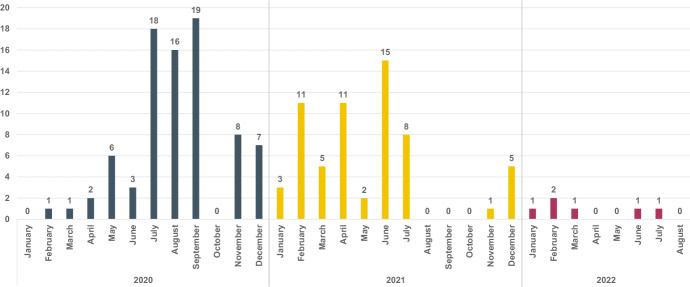
Moratoria issued. Source: Surveilligence, s.r.o. (data provided to the author on a private basis)

One of the novelties introduced during the pandemic was an extraordinary moratorium. The extraordinary moratorium was supposed to be a tool that would provide entrepreneurs on the verge of insolvency due to extraordinary events with some sort of breathing space. It was based on the provisions of the then (ordinary) moratorium. In both the pre-pandemic and the post-pandemic era roughly one ordinary moratorium was issued each month on average, as can be seen from the statistics shown in Fig. 3 above. The extraordinary moratorium was used more often. Still, it was used much less than I initially expected when I was involved in the drafting of the relevant provision in April 2020. Its limited use was probably due primarily to its public nature and potential stigma (the respective court decision being publicly available in the insolvency register) and to the enactment of the option to suspend loan payments.

## Suspension of Loan Repayments

**Fig. 4:**
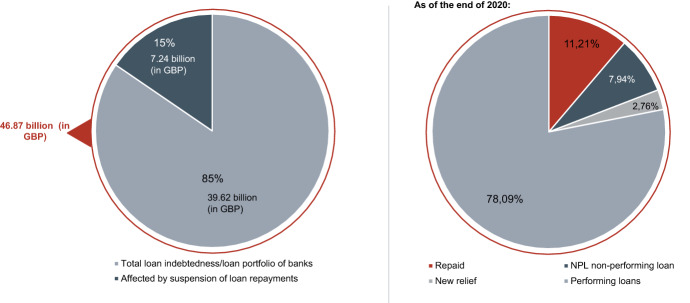
Loan repayments. Source: Data provided by the Czech National Bank, with individual edits made by the author

As can be seen from Fig. 4, a considerable number of entrepreneurs asked for suspension of their loan repayments. Overall, approximately 15% of the whole loan portfolio of local banks regulated by the Czech National Bank were affected by the suspension.^6^ The Czech National Bank overseeing relevant banks in the Czech Republic concluded that the measure was useful and without significant side effects. As of the end of 2020, less than 8% of those affected loans were registered as so-called non-performing loans.^7^ The vast majority of affected loans were registered as performing loans without any other relief provided to debtors.^8^

## Future Outlook

Looking forward, we are facing difficult challenges. Household confidence seems to be at the lowest level since 2002, when it was first assessed.^9^ Inflation and borrowing costs have been spiking.^10^ As in other parts of the world, entrepreneurs encounter various difficulties ranging from supply chain constraints to staff shortages. Good news is that the level of non-performing loans is still at a low level.^11^

In this context, I would like to make a couple of comments. First of all, it appears that businesses are seeking formal insolvency solutions too late.^12^

Secondly, the Czech Republic has, unfortunately, not yet implemented the EU Directive on Restructuring and Insolvency.^13^ Therefore, Czech law does not provide for any complex framework of preventive restructuring that would give proper incentives to deal with financial distress at lower costs compared to a more formal and burdensome insolvency process.

Thirdly, even in the absence of any preventive restructuring regime, I would reject calls for a revival of the extraordinary moratorium. Different from the COVID-19 crisis, the current situation has not arisen so unexpectedly. Of course, we could not foresee Russia’s war with Ukraine. However, the current energy crisis is still following a steady and predictable progression. As such, there is a time for a well-thought and structured approach.

## Footnotes

1 Lasak (2023).

2 Schönfeld et al. (2019).

3 Act No. 191/2020 Coll. on certain measures to mitigate the effects of the SARS CoV-2 coronavirus epidemic on parties participating in court proceedings, aggrieved parties, victims of crime and legal entities and on the amendment of the Insolvency Act and the Code of Civil Procedure.

4 Act No. 177/2020 Coll. on certain pandemic-related loan repayment measures (‘Lex Covid’).

5 Figure 1 was prepared by the author.

6 Data provided by the Czech National Bank, with individual edits made by the author.

7 Czech National Bank (2021).

8 Ibid.

9 Czech Banking Association (2022).

10 Ibid.

11 Czech National Bank (2022).

12 Schönfeld et al. (2019).

13 Directive (EU) 2019/1023 of the European Parliament and of the Council of 20 June 2019 on preventive restructuring frameworks, on discharge of debt and disqualifications, and on measures to increase the efficiency of procedures concerning restructuring, insolvency and discharge of debt, and amending Directive (EU) 2017/1132 (Directive on restructuring and insolvency).
